# Complete mitochondrial DNA sequence of the invasive hornet *Vespa velutina* (Insecta, Hymenoptera) found in Japan

**DOI:** 10.1080/23802359.2017.1289353

**Published:** 2017-03-21

**Authors:** Ryoichi Takahashi, Hisashi Okuyama, Takuya Kiyoshi, Jun-ichi Takahashi

**Affiliations:** a Department of Life Sciences, Kyoto Sangyo University, Kyoto, Japan;; b Department of Zoology, National Museum of Nature and Science, Tokyo, Japan

**Keywords:** Asian hornet, illumina sequencing, genetic distance, alien species, invasive species

## Abstract

In this study, we analyzed the complete mitochondrial genome of the invasive Asian hornet *Vespa velutina* from Japan. The mitochondrial genome of *V. velutina* was identified as a circular molecule of 16,765 bp, similar to that in other hornet species. It was predicted to contain 13 protein-coding, 20 tRNA, and two rRNA genes, along with one A + T-rich control region. The initiation codons ATC was found in one, ATG in four, ATT in five, and ATA in three genes, while TAA was the termination codon in all these genes. The average AT content of 13 protein-coding was 82%.

The naturalization of the invasive Asian hornet *Vespa velutina* has resulted in general decline of the native hornet population and apiculture and increase in sting injuries across non-native countries, including South Korea, Japan, and countries in Europe (Choi et al. 2012; Monceau et al. 2014). In Japan, *V. velutina* was first observed on Tsushima Island in 2012 (Sakai & Takahashi 2014). Tsushima Island is an important region in the ecosystems of the Japanese Archipelago. To conduct an effective prevention of *V. velutina* introduction and establishment, genetic data are necessary to identify the invasive path of this species.

Here, we report the complete mitochondrial genome of *V. velutina*, which will enhance our knowledge on its invasion routes in Japan and thus help its extermination. Adult worker was collected from the front of a honeybee hive located on Tsushima Island, Nagasaki Prefecture, Japan (the specimen was stored in the National Museum of Nature and Science, Japan, accession number: NSMT-I-HYM 74241). Genomic DNA isolated from one worker was sequenced using Illumina’s Next Seq 500 (Illumina, USA). The resultant reads were assembled and annotated using the MITOS web server (Bernt et al. 2013), MEGA6 (Tamura et al. 2013), and GNETYX v.10 (Genetyx Corporation, Japan). The phylogenetic analysis was performed using TREEFINDER version (Denmark) of March 2011 (Jobb et al. 2004) based on the nucleotide sequences of 13 protein-coding genes (Figure 1).

**Figure 1.**
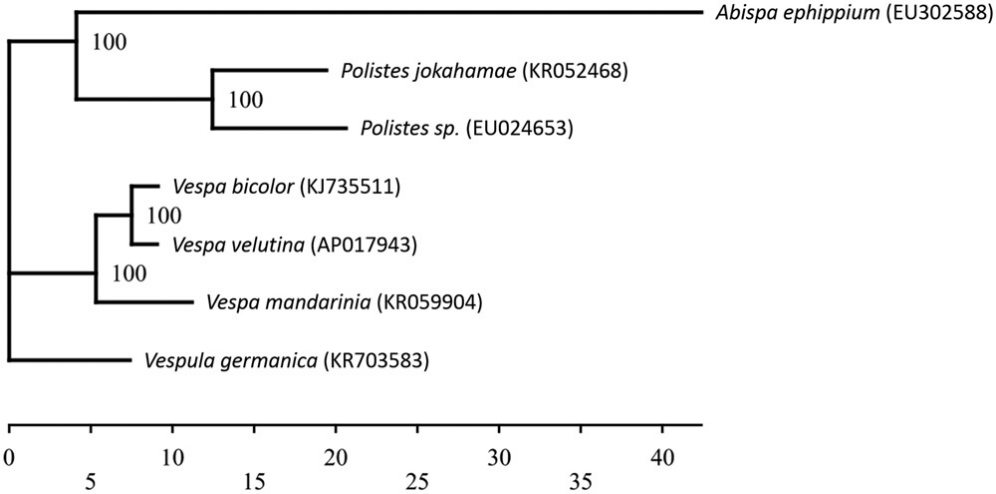
Phylogenetic relationships (maximum likelihood) of the Vespidae based on the nucleotide sequence of 13 protein-coding genes of the mitochondrial genome. The numbers at the nodes indicate bootstrap support inferred from 1000 bootstrap replicates. The sequence of *Abispa ephippium* was used as outgroup. Alphanumeric terms indicate the GenBank accession numbers.

The *V. velutina* mitochondrial genome forms a 16,765 bp-long closed loop (accession number AP017943). It represents a hornet mitochondrial genome and matches the genomic organization common in *V. velutina* in that it comprises 13 protein-coding, 20 putative tRNA, and two rRNA genes, as well as an A + T-rich control region. The average AT content of the *V. velutina* mitochondrial genome was 81.9%. Similar to other hornet mitochondrial genomes, the heavy strand was predicted to have nine protein-coding and 13 tRNA genes and the light strand was predicted to contain four protein-coding, seven tRNA, and two rRNA genes. The genes *ND4* and *ND4L* shared seven nucleotides. Of the 13 protein-coding genes, the initiation codons ATC was found in one, ATG in four, ATT in five, and ATA in three genes, while TAA was the termination codon in all these genes. Phylogenetic analysis using the 13 mitochondrial protein-coding genes from seven closely related taxa of Vespidae (Cameron et al. 2008; Song et al. 2016; Wei et al. 2016; Zhou et al. 2016) suggested a sister relationship between *V. velutina* and *V. bicolor* (Archer 2012; Chen et al. 2016).

A previous partial sequence analysis of *V. velutina* mitochondrial DNA provided a rough estimate of the invasion route of this species to Europe and East Asia (Perrard et al. 2013; Takeuchi et al. 2017), but it was not able to analyze the origin and population genetic structure. The complete mitochondrial DNA data of *V. velutina* will help to develop specific primers for identification of strains and estimation of genetic diversity. The sequence analysis of the *V. velutina* mitochondrial genome provides important information about environmental adaptation and origin of invasive strains.
